# Epidemiology of Macrosomia in Saudi Arabia: An Analysis of 12,045 Pregnancies from the Riyadh Mother and Baby Multicenter Cohort Study (RAHMA) Database

**DOI:** 10.3390/healthcare12242514

**Published:** 2024-12-12

**Authors:** Hayfaa Wahabi, Amel Fayed, Nouran Aleyeidi, Samia Esmaeil

**Affiliations:** 1 Research Chair for Evidence-Based Health Care and Knowledge Translation, King Saud University, Riyadh 11451, Saudi Arabia; hwahabi@ksu.edu.sa (H.W.); sesmaeil@ksu.edu.sa (S.E.); 2 Department of Family and Community Medicine, College of Medicine, King Saud University, Riyadh 11451, Saudi Arabia; 3 Department of Family and Community Medicine, College of Medicine, Princess Nourah Bint Abdulrahman University, Riyadh 11671, Saudi Arabia; naaleyeidi@pnu.edu.sa

**Keywords:** macrosomia, pregnancies, epidemiology, Saudi Arabia

## Abstract

Objective: To investigate the prevalence, risk factors, and complications associated with delivering macrosomic babies. Methods: Singleton term pregnancies (12,045) were studied. Macrosomia was categorized using the following two definitions: birthweight > 4 kg and birthweight ≥ 90th percentile (3.7 kg). Regression models were developed to identify significant risk factors for macrosomia such as maternal age, parity, pre-pregnancy body mass index, gestational weight gain, and hyperglycemia. Other models were constructed to identify the independent effect of macrosomia on outcomes such as shoulder dystocia, emergency cesarean section, stillbirth, and low APGAR scores. Results: The 50th centile birth weight of Saudi term infants is 3.18 kg; the 90th and 95th centiles were 3.70 and 3.91 kg, respectively. The prevalence of macrosomia (>4 kg) was 3.4%. The likelihood of macrosomia was associated with maternal age >40 years for newborns >4 kg, OR = 1.88, 95% CI (1.02–3.48), and maternal age < 18 for newborns ≥90th centile, OR = 5.23, 95% CI, (1.05–26.06). Regardless of the classification of macrosomia, it was associated with gestational age ≥41 weeks, parity > 4, pre-pregnancy BMI > 30, and maternal hyperglycemia. Macrosomia, using either definition, was associated with increased risk of shoulder dystocia, OR = 11.45, 95% CI (4.12–31.82) and OR = 9.65, 95% CI (3.89–23.94), and emergency CS, OR = 2.03, 95% CI (1.36–3.08) and OR = 1.77, 95% CI (1.34–1.52), for birthweight > 4 kg and ≥90th centile, respectively. Furthermore, newborns whose weights >4 kg were at greater risk to be stillborn, OR = 4.24, 95% CI (1.18–15.20), and to have low APGAR scores at birth, OR = 3.69, 95% CI (1.25–10.98). Conclusion: The risk of macrosomia among Saudi women significantly increases with maternal age, parity, gestational age, hyperglycemia, and pre-pregnancy obesity. Regardless of the definition used, delivering a macrosomic baby was associated with risks of shoulder dystocia and emergency cesarean section. Newborns (>4 kg) were at greater risk of stillbirth and low APGAR scores.

## 1. Introduction

Macrosomia is commonly defined by researchers as a birth weight greater than 4000 g at term [1,2,3]. This definition, which is based on crude birthweight, is appropriate to examine the effects of the baby’s size on the progression of labor and delivery and possible complications to the mother and baby. However, to encompass the additional effects of birthweight on the outcomes of pregnancy, a wider definition of large for gestational age (LGA) and small for gestational age (SGA), has been adopted by other researchers, which corresponds to the birthweight >90 centile and <10 centile for the gestational age, respectively [4,5,6]. The prevalence of LGA varies with gestational age, sex of the fetus, and ethnicity [7]. The epidemiology of LGA has not been investigated yet in Saudi Arabia; however, there are a few published reports on the prevalence and risk factors for macrosomia based on birthweight ≥4000 g [8].

There is significant variation in the prevalence of macrosomia >4000 g globally; a recently published systematic review reported a wide range from 0.9 to 29.8% [9]. In Saudi Arabia, the prevalence of macrosomia ≥4000 g is 3.4%; however, it is much higher among women with gestational diabetes (GDM) and pre-gestational diabetes (pre-GDM), as recent reports showed a prevalence range from 10 to 19.8% [10,11].

Maternal characteristics, such as pre-pregnancy weight, gestational weight gain (GWG), maternal age, parity, and metabolic status, consistently influence fetal weight. However, the degree to which each of these factors affects birth weight varies across populations [12].

Macrosomia is associated with well recognized maternal, fetal, and neonatal adverse outcomes. Most of these complications are associated with the large fetal size navigating the birth canal, causing maternal trauma such as obstructed labor and ruptured uterus, perineal tear, and anal sphincter rupture [9]. In addition, the macrosomic fetus is at greater risk of birth trauma from shoulder dystocia or intrauterine fetal death from fetal asphyxia [13,14,15]. Macrosomic neonates are at a greater risk of admission to NICU due to birth trauma, birth asphyxia, hypoglycemia, or transient tachypnea of the newborn [9]. Furthermore, many of these infants have an increased predisposition to obesity and diabetes in adult life [16,17].

A few recent studies from Saudi Arabia have explored various aspects of pregnancies complicated by the delivery of macrocosmic infants, including prevalence, risk factors, and associated outcomes. However, most of these studies included either a small sample size [11] or investigated macrosomia associated with certain maternal risk factors such as obesity or GDM [18,19].

The objective of this study is to examine the epidemiology of macrosomia in Saudi Arabia, including its prevalence, maternal risk factors, and the maternal and neonatal complications associated with the delivery of a macrosomic infant. This will be achieved using the data from the Riyadh Mother and Baby Multicenter Cohort Study (RAHMA). In addition, we aim to investigate the differences between absolute and centile birthweight with respect to risk factors and complications.

## 2. Methods

### 2.1. Study Design, Setting, and Eligibility Criteria

Saudi Arabia has a rapidly growing population (approaching 38 M), with rapid urbanization (85% of population is urban) and high rates of both maternal obesity and gestational diabetes, which significantly impact birth weight outcomes. Furthermore, the cultural preference for large families and specific nutritional practices contribute to diverse birth weight trends. Riyadh is the capital city of Saudi Arabia and serves as the administrative center of the Riyadh Region. The Riyadh Region is one of Saudi Arabia’s 13 administrative provinces and is the largest in terms of population (7.82 M). This makes it the largest city in Saudi Arabia by population (nearly 20%), highlighting its role as the country’s political, economic, and cultural hub.

The Riyadh mother and baby multicenter cohort study (RAHMA) is a large multicenter cohort study. The main objective of the study was to investigate the epidemiology of the common adverse pregnancy outcomes in Saudi Arabia. The RAHMA database includes the data of 14,500 Saudi pregnant women and their infants collected from three tertiary maternity hospitals in Riyadh.

The RAHMA cohort recruitment began in three selected hospitals in Riyadh. Hospitals were chosen using stratified cluster random sampling, based on hospital type, including Ministry of Health (MOH), military, and university hospitals. Private hospitals were excluded due to logistical constraints and their low delivery rates.

Information on the socioeconomic status and antenatal history of the women in the study was collected through the completion of a self-administered questionnaire. Additionally, obstetric and laboratory data of the participants’ medical records were added to the database of the study. Further details of the RAHMA study design can be found in Wahabi et al. [3].

To avoid possible selection and reporting biases, all women fulfilling the eligibility criteria from the participating hospitals were invited to participate in the study. Pre-structured questionnaires were self-administered to participating women to collect sociodemographic variables while all obstetric-related data were collected via abstracting electronic medical records by a standard data-collection sheet. Immediate outcomes were reported in the current study and no loss of follow-up was defined.

For the current study, participants who fulfilled the following criteria were included:

1. Gestational age of 37 weeks or more at the time of delivery, calculated according to the last menstrual period and/or the earliest fetal ultrasound.
2. Singleton pregnancy.
3. Documented birthweight.

### 2.2. Sample Size Calculation

The minimum sample size required in the current study was 9172, considering a power of 99%, alpha of 0.01, and a prevalence as low as 1% with a margin of error of ±1%. STATA version 16 was used for sample size estimation.

Definitions:

1. Gestational age was calculated from the last menstrual period and/or the first-trimester ultrasound scan [20].
2. Term was defined as gestational age ≥37 weeks.
3. Birthweight was the weight of the newborn to the nearest gram.
4. Shoulder dystocia was defined as a vaginal delivery requiring an additional obstetric maneuver to deliver the fetus after delivery of the head and failure of gentle traction [21].
5. Stillbirth was defined as a birth of a fetus with no signs of life ≥24 weeks gestation.
6. Maternal body mass index (BMI) was calculated from weight prior to pregnancy and height measured during the first antenatal clinic. Participants were classified according to the WHO BMI definitions as follows: underweight, ≤18.4 kg/m^2^; healthy weight, 18.5–24.9 kg/m^2^; overweight, 25.0–29.9 kg/m^2^; and obese, ≥30 kg/m^2^ [22].
7. Gestational Weight gain (GWG) was calculated as the difference between the term weight and the pre-pregnancy weight.
8. Gestational diabetes mellitus (GDM) was diagnosed at any time in pregnancy according to World Health Organization guidelines if one or more of the following criteria were met [23]:
  - Fasting plasma glucose 5.1–6.9 mmol/L (92–125 mg/dL).
  - Plasma glucose ≥ 10.0 mmol/L (180 mg/dL) one hour following a 75 g oral glucose load.
  - Plasma glucose 8.5–11.0 mmol/L (153–199 mg/dL) two hours following a 75 g oral glucose load.
9. Pre-gestational diabetes mellitus (pre-GDM) is a condition in which the mother has diabetes (most commonly type 1 or type 2 diabetes) before the onset of pregnancy [24]. For this study, all cases who developed GDM or had pre-GDM were grouped together as hyperglycemia.
10. Hypertensive events during pregnancy according to the report of the national high blood pressure [25] included pre-eclampsia, which is defined as the new onset of elevated blood pressure after 20 weeks of pregnancy in a previously normotensive woman in addition to proteinuria of at least 1+ on a urine dipstick or ≥300 mg in a 24 h urine collection. For this study, and due to the low prevalence of these conditions, all cases were aggregated in one category.
11. Second-hand smoking (SHS) refers to exposure to tobacco smoke by a non-smoker, typically in a household setting. In the context of this study, SHS exposure is defined as a situation where a woman who did not smoke during pregnancy lived with a household member (such as a husband, son, daughter, or other relatives) who smoked during the pregnancy.

### 2.3. Statistical Analysis

Macrosomia was considered according to the following two different definitions: crude birthweight exceeding 4000 g and those with birthweight equals to or exceeding the 90th percentile (3700 g as measured from the studied the population). The results are expressed as percentages representing the proportion of women in each birthweight category, with the chi-square test utilized for the initial assessment of the association between having macrosomic infants and various risk factors. Logistic regression models were developed to evaluate the independent influence of potential risk factors on the likelihood of having a macrosomic infant, considering the normal birth weight ranges as the reference group (babies with birthweights ≤4000 g and those within the 10th to 90th centiles). Adjustment of clinically significant risk factors such as maternal age, parity, gestational age, gestational weight gain, and maternal hyperglycemia during pregnancy were considered.

Additionally, multiple logistic regression models were constructed to assess the independent impact of macrosomia on neonatal and maternal outcomes such as shoulder dystocia, perineal tear, mode of delivery, admission to intensive care units, stillbirth, and low APGAR scores. These models were adjusted for maternal age, pregnancy BMI, gestational age, parity, diabetes, and hypertension.

The findings were presented as Odds Ratios (OR) along with their corresponding 95% Confidence Intervals (CI). For improving the robustness of the regression model, bias correction using the Firth procedure was applied for the logistic regression of rare events.

All statistical tests utilized a significance level of *p* < 0.05. As the recruited sample size exceeded the minimal sample size required, no imputation of missing data was applied and missing data were excluded from the analysis at each variable level.

## 3. Results

**Figure 1:**
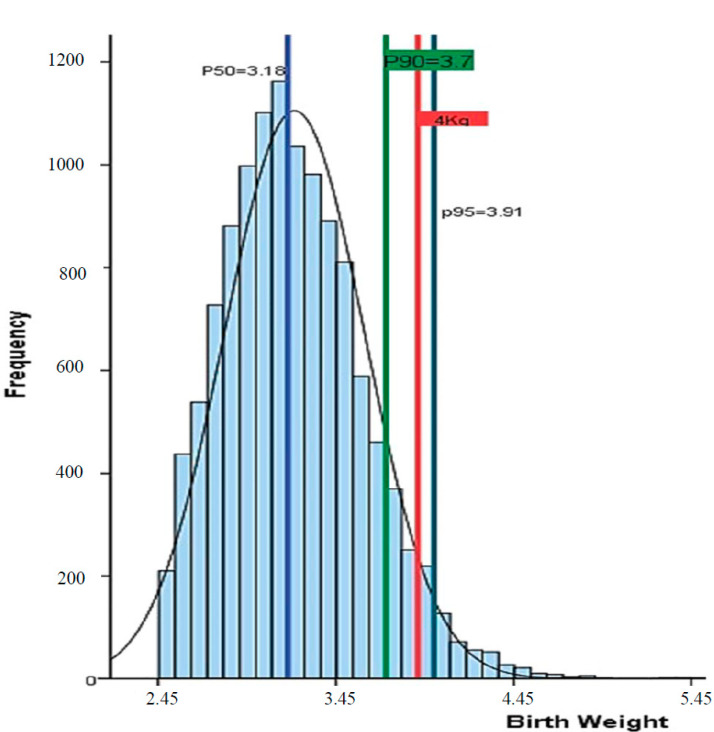
Distribution of birthweight (in kg) in the studied sample with the definition of different percentiles.

A total of 12,045 singleton pregnancies from the RAHMA database were analyzed. The normal distribution of the birthweight of neonates in the cohort (Figure 1) shows that the 50th centile of birthweight of Saudi infants born at term is 3.18 kg, the 90th centile is 3.70 kg, and the 95th centile is 3.91 kg. The prevalence of macrosomia >4 kg was 3.4%.

Predisposing factors and pregnancy outcomes were compared in groups of birthweights 2.5–4 kg (n = 11,644) and >4 kg (n = 410), and by centile birthweight 10th–90th centile (n = 9685) and >90th centile (n = 1194). The demographic characteristics of the groups are reported in Table 1. The prevalence of macrosomia significantly increases with the increase in mother’s age, parity, gestational age ≥ 41 weeks, and diabetes. In addition, it increases with maternal pre-pregnancy obesity and with excessive gestational weight gain.

Pregnancy outcomes for macrosomia adjusted for maternal age, parity, pre-pregnancy BMI, HTN, and hyperglycemia are provided in Table 2 and Table 3. In this study, macrosomia, using either definition, was associated with an increased risk of shoulder dystocia, OR = 11.45, 95% CI (4.12–31.82) and OR = 9.65, 95% CI (3.89–23.94), and emergency CS, OR = 2.03, 95% CI (1.36–3.08) and OR = 1.77, 95% CI (1.34–1.52), for birthweight > 4 kg and >90th centile, respectively. Furthermore, newborns whose weights >4 kg were at greater risk to be stillborn, OR = 4.24, 95% CI (1.18–15.20), and to have low APGAR scores at birth, OR = 3.69, 95% CI (1.25–10.98). However, mothers of macrosomic newborns in the current study were not at increased risk of perineal tears, induction of labor, or admissions to intensive care units (Table 2 and Table 3).

Analysis of predisposing factors for birthweight >4 kg and >90th centile (Table 4) revealed that the likelihood of macrosomia was associated with maternal age >40 years for macrosomic newborns >4 kg, OR = 1.88, 95% CI (1.02–3.48), and with maternal age <18 for macrosomic newborns >90th centile, OR = 5.23, 95% CI, (1.05–26.06). In addition, macrosomia was associated with gestational age ≥41 weeks, OR = 1.55, 95% CI (1.29–1.87), and OR = 1.50, 95% CI (1.34–1.69), for macrosomia defined as birthweight >4 kg and >90th centile, respectively; with parity >4, OR = 2.42, 95% CI (1.28–4.58), and OR = 1.47, 95% CI, (1.03–2.06), for macrosomia defined as birthweight >4 kg and >90th centile, respectively; and with pre-pregnancy BMI >30, OR = 3.66, 95% CI (2.05–6.54), and OR = 1.9, 95% CI (1.43–2.53), for macrosomia defined as birthweight >4 kg and >90th centile, respectively; in addition to maternal hyperglycemia during pregnancy, OR = 1.53 (1.09–2.15), and OR = 1.38, 95% CI (1.11–1.70) for macrosomia defined as birthweight >4 kg and >90th centile, respectively.

Pre-pregnancy BMI > 30 kg/m^2^ was associated with the greatest risk factor for birthweight >90th centile and for birthweight >4 kg, while inadequate weight gain during pregnancy and pre-pregnancy BMI < 20 kg/m^2^ significantly attenuated the risk of having birth weights >90th centiles.

## 4. Discussion

This study is the largest to assess the epidemiology of macrosomic pregnancy in Saudi Arabia. It shows that the 50th centile of birthweight of Saudi infants born at term is 3.18 kg, the 90th centile is 3.70 kg, and the 95th centile is 3.91 kg. The prevalence of macrosomia (birth weight > 4 kg) was found to be 3.4%. After adjusting for confounding factors, several maternal risk factors were significantly associated with an increased risk of macrosomia among Saudi women. These include maternal age over 40 years, a parity of more than four, gestational age of 41 weeks or more, and maternal hyperglycemia. Irrespective of macrosomia definition, in this study, the delivery of a macrosomic baby was associated with an increased risk of shoulder dystocia and emergency CS. Moreover, newborns whose weights were >4 kg were at greater risk of stillbirth and having low APGAR scores at birth. Mothers of macrosomic newborns in the current study were not at an increased risk of perineal tears, induction of labor, or admissions to intensive care units.

The fact that both the 90th and the 95th centile of birthweight of at term infants is <4 kg indicates the unique profile of the birthweight in the Saudi population. Normal variations in birthweight across the globe have been documented by many studies [4,6,26]. Known factors, such as maternal characteristics and fetal sex, did not account for the wide range of normal birthweights observed among women from different geographical areas, which indicates the significant effect of ethnicity, culture, and geographical area on the birthweight of a specific population [27].

The interrelated maternal characteristics associated with the delivery of a macrosomic baby in this study were similar to those reported by other investigators [12,28]. Previous studies showed that GDM and pre-existing diabetes are both associated with fetal macrosomia [29,30,31,32]. Obesity [8] and older maternal age are in the causal pathway of peripheral insulin resistance, leading to maternal hyperglycemia, hence the abundance of glucose influx to the fetus and fetal overgrowth [33]. Preconception control of pre-GDM [34] and maternal obesity [8], in addition to the monitoring and controlling of GWG [35] of obese women, may be effective strategies to reduce the prevalence of macrosomic pregnancies and the associated complications among Saudi mothers.

We were not able to explain the association between maternal age <18 years and the delivery of a macrosomic baby in the absolute birthweight analysis; however, similar results were reported by other investigators [12].

Prolonged labor, instrumental delivery, and CS are complications reported in association with the delivery of macrosomic fetuses [36,37,38]. In this study, shoulder dystocia and emergency CS were reported significantly more frequently among macrosomic compared to normal fetal weight pregnancies (Table 2 and Table 3). However, it is noticeable that the risks were greater when analyzed by birthweight >4 kg, indicating that these outcomes are directly related to fetal size (Table 2 and Table 3), which agrees with the results of a recently published systematic review [9]. In addition, pregnancies with fetal weight >4 kg had a fourfold risk of being stillborn and more than a threefold increased risk of low APGAR scores at birth compared to normal weight fetuses (Table 2). The association between macrosomia and stillbirth were reported in previous published studies [28,39]; similarly, other authors reported an increased risk of fetal asphyxia, low APGAR scores, and increased admission to neonatal special care units [12,38,40].

The increased fetal and neonatal mortality and morbidity associated with macrosomic pregnancy can be attributed to the increased risk of prolonged labor and birth injuries [38], in addition to the complications of maternal hyperglycemia and obesity [41,42]. Hence, it is prudent to consider pregnancy with risk factors for macrosomia as high-risk and to include pre-birth fetal weight estimation and a carefully planned mode of delivery in the management of such women [43].

In this study, we did not find any association between the delivery of a macrosomic infant and some maternal complications such as perineal tears. These results are not surprising considering that these complications are related to fetal size and that only 3.4% of the infants in this study had a birthweight >4 kg, especially when considering that the outcomes of macrosomic pregnancies vary with the characteristics of the population examined and the spectrum of the birthweight of the macrosomic pregnancies [9].

We believe that this study has many strengths; as this study is the largest birth cohort (12,045 pregnancies) to investigate the epidemiology of macrosomia in Saudi Arabia, it examined macrosomia using two different definitions, >4 kg and ≥90th percentile, with different outcomes for each definition. However, we acknowledge the limitations of this study, as it has not examined all the possible complications and outcomes of macrosomic pregnancies, such as postpartum hemorrhage and the duration of labor, due to a lack of data.

## 5. Conclusions

The risk of macrosomia among Saudi women significantly increases with maternal age, parity, gestational age, maternal hyperglycemia, and pre-pregnancy obesity. Regardless of the definition used, delivering a macrosomic baby is associated with increased risks of shoulder dystocia and emergency cesarean sections. Moreover, newborns whose weights are >4 kg are at greater risk of stillbirth and low APGAR scores at birth. There is a need for national clinical guidelines for the management of pregnant women at risk of delivering a macrosomic infant.

## Figures and Tables

**Table 1:** Demographic characteristics of women per birthweight group.

		Birth Weights Categories					
		2.5–4 kg (n = 11,644)	>4 kg (n = 410)	*p*-Value	10th–90th Centile (n = 9685)	≥90th Centile (n = 1194)	*p*-Value
		%	%		%	%	
Total prevalence of birtheight categories		(96.6)	(3.4)		(80.3)	(9.9)	-
Maternal age (years)	<18	(0.3)	(0.0)		(0.3)	(0.2)	<0.01
	18–34	(76.2)	(63.8)		(76.5)	(68.0)	
	35–40	(19.3)	(28.9)		(19.1)	(25.6)	
	>40	(4.2)	(7.3)		(4.1)	(6.2)	
Working status	Housewife	(87.6)	(83.4)	0.02	(87.5)	(87.3)	0.90
	Employee/Student	(12.4)	(16.6)		(12.5)	(12.7)	
Educational level	Illiterate	(2.9)	(4.2)	0.28	(3.0)	(3.4)	0.53
	School	(56.4)	(52.1)		(56.5)	(54.4)	
	University and above	(40.6)	(43.7)		(40.6)	(42.2)	
Parity	P2–P4	(48.1)	(44.5)	<0.01	(48.4)	(48.2)	<0.01
	Primiparous	(21.7)	(10.3)		(21.5)	(13.1)	
	P5+	(30.2)	(45.2)		(30.1)	(38.7)	
Prepregnancy BMI (kg/m^2^)	<20	(4.9)	(1.0)		(4.8)	(1.1)	<0.01
	20–24.9	(25.4)	(11.4)		(25.5)	(17.0)	
	25–29.9	(33.9)	(27.2)		(34.1)	(30.2)	
	30+	(35.8)	(60.4)		(35.6)	(51.7)	
Gestational Weight Gain	Inadequate	(31.9)	(27.0)	0.03	(32.2)	(24.4)	<0.01
	Adequate	(34.4)	(30.5)		(34.1)	(33.5)	
	Excessive	(33.7)	(42.5)		(33.7)	(42.1)	
Second-hand smoking	No	(77.1)	(79.7)	0.27	(77.2)	(78.2)	0.51
	Yes	(22.9)	(20.3)		(22.8)	(21.8)	
Gestational age (weeks)	Full term (37–40)	(81.9)	(68.3)	<0.01	(82.8)	(71.5)	<0.01
	41 weeks and more	(14.2)	(28.8)		(14.2)	(26.1)	
Hypertension		(2.2)	(2.7)	0.53	(2.2)	(2.4)	0.55
Diabetes mellitus		(28.0)	(44.0)	<0.01	(27.9)	(37.8)	<0.01

BMI: Body Mass Index. All data are expressed as percentages.

**Table 2:** Maternal and neonatal outcomes associated with birthweight >4 kg.

	2.5–4 kg (n = 11,644)	>4 kg (n = 410)	OR (95% C.I.)	*p*-Value
	%	%		
Shoulder dystocia	(0.3)	(5.0)	11.45 (4.12–31.82) *	<0.01
Perineal tear	(0.3)	(0.5)	3.22 (0.39–26.09)	0.27
Induction of labor	(15.8)	(17.1)	1.22 (0.82–1.83)	0.32
Instrumental delivery	(4.3)	(2.9)	1.45 (0.61–3.47)	0.76
Elective CS	(9.5)	(11.0)	0.69 (0.38–1.28)	0.14
Emergency CS	(12.8)	(22.4)	2.03 (1.36–3.08) *	<0.01
Maternal admission ICU	(0.3)	(0.5)	3.79 (0.45–32.24)	0.22
Neonatal admission to NICU	(2.2)	(4.2)	1.24 (0.45–3.48)	0.67
Stillbirth	(0.5)	(1.7)	4.24 (1.18–15.20) *	0.03
APGAR < 7 at 5 min	(0.7)	(2.9)	3.69 (1.25–10.98) *	0.02

OR: Odds Ratio; C.I.: Confidence Interval; ICU: Intensive Care Unit; NICU: Neonatal Intensive Care Unit. CS: Cesarean Section. All regression models were adjusted for maternal age, parity, pre-pregnancy body mass index, hypertension, and hyperglycemia. * *p* < 0.05.

**Table 3:** Maternal and neonatal outcomes associated with birthweight ≥90th centile.

	10th–90th Centile (n = 9685)	≥90th Centile (n = 1194)	OR (95% C.I.)	*p*-Value
	%	%		
Shoulder dystocia	0.2	2.7	9.65 (3.89–23.94) *	<0.01
Perineal tear	0.3	0.4	1.61 (0.35–7.36)	0.54
Induction of labor	15.5	18.0	1.16 (0.90–1.49)	0.25
Mode of delivery				
Instrumental delivery	4.4	2.9	1.19 (0.70–1.62)	0.67
Elective CS	9.1	11.5	1.17 (0.84–1.63)	0.54
Emergency CS	12.3	17.8	1.77 (1.34–1.52) *	<0.01
Maternal admission ICU	0.3	0.3	1.11 (0.13–9.18)	0.93
Neonatal admission to NICU	2.0	3.0	1.05 (0.52–2.14)	0.89
Stillbirth	0.5	0.8	2.03 (0.66–3.57)	0.22
APGAR < 7 at 5 min	0.7	1.5	2.02 (0.81–5.08)	0.13

OR: Odds Ratio; C.I.: Confidence Interval; ICU: Intensive Care Unit; NICU: Neonatal Intensive Care Unit; CS: Cesarean section. All regression models were adjusted for maternal age, parity, pre-pregnancy body mass index, hypertension, and hyperglycemia. * *p* < 0.05.

**Table 4:** Multivariate analysis for risk factors for delivery of macrosomic babies.

	>4 kg (N = 410)	≥90th Centile (N = 1194)
Age (years)		
<18	-	5.23 (1.05–26.06) *
18–34	1	1
35–40	1.07 (0.70–1.65)	1.20 (0.92–1.57)
>40	1.88 (1.02–3.48) *	1.48 (0.94–2.34)
Gestational age		
Full term	1	1
≥41 weeks	1.55 (1.29–1.87) *	1.50 (1.34–1.69) *
Parity		
Primiparous women	1	1
Para 2–4	1.80 (1.0–3.27)	1.34 (0.99–1.81)
Para > 4	2.42 (1.28–4.58) *	1.47 (1.03–2.06) *
Pre-pregnancy BMI (kg/m^2^)		
<20	0.44 (0.05–3.35)	0.38 (0.15–0.95) *
20–24	1	1
25–30	1.89 (1.01–3.55) *	1.18 (0.87–1.60)
>30	3.66 (2.05–6.54) *	1.9 (1.43–2.53) *
Gestational weight Gain		
Appropriate	1	1
Excessive	1.34 (0.94–2.06)	1.26 (0.99–1.59)
Inadequate	0.81 (0.53–1.23)	0.66 (0.51–0.86) *
Hyperglycemia	1.53 (1.09–2.15) *	1.38 (1.11–1.70) *

BMI: Body Mass Index. Regression models adjusted for maternal age, gestational age, parity, pre-pregnancy BMI, gestational weight gain, and hyperglycemia. * *p* < 0.05.

## Data Availability

Data from this study are available to researchers upon request and approval of the Institutional Review Board at Princess Nourah Bint Abdulrahaman University (irb@pnu.edu.sa). The request and approval of data sharing are independent of the research team.
